# Exploring the alignment between paramedicine's professional capabilities and competency frameworks for current and evolving scopes of practice: a literature review

**DOI:** 10.1186/s12909-023-04992-w

**Published:** 2024-01-05

**Authors:** Anthony Weber, Scott Devenish, Louisa Lam

**Affiliations:** 1 School Business and Law | Higher Education Division, CQUniversity Australia, Building 34/2.26, Bruce Highway, Rockhampton, QLD 4701 Australia; 2 School of Nursing, Midwifery and Paramedicine, Faculty of Health, Australian Catholic University, Brisbane, Australia; 3 School of Nursing, Midwifery and Paramedicine, Faculty of Health, Australian Catholic University, Melbourne, Australia; 4 School of Public Health and Preventive Medicine, Faculty of Medicine Nursing and Health Sciences, Monash University, Melbourne, Australia; 5 Institute of Health and Wellbeing, Federation University Australia, Ballarat, Australia

**Keywords:** Paramedicine, Competencies, Competency frameworks, Professional capabilities, Accreditation, Evolving scopes of practice

## Abstract

**Background:**

To adequately prepare graduates for the dynamic demands of paramedic practice, adopting a contemporary educational approach is essential. This involves collaborating to identify crucial competencies through input from industry stakeholders, experienced practitioners, and discipline-specific experts. Accreditation assumes a central role within this framework, serving as a cornerstone to ensure that paramedicine curricula align with paramedics' diverse and evolving professional roles.

**Methods:**

A narrative review of the literature and a directed search of grey literature were performed to identify specific developments in paramedicine competencies and scope of practice and mapped to the professional capabilities published by the Paramedicine Board of Australia. In determining a competency map and accreditation’s role in a competency framework specific to current and evolving paramedic practice, key documents were analysed using a qualitative approach based on content analysis to identify common traits among documents, countries and other professions.

**Results:**

The review process identified 278 themes that were further allocated to 22 major analytical groupings. These groupings could further be mapped to previously reported cognitive, technical, integrative, context, relationship, affective/moral competencies and habits of mind. At the same time, the highest-rated groupings were key competencies of intellectual skills, safety, accountability, clinical decision-making, professionalism, communications, team-based approach and situational awareness. Two groups were represented in the literature but not in the professional capabilities, namely Health and Social continuum and self-directed practice.

**Conclusions:**

This review highlights the importance of measuring and validating the professional capabilities of Paramedicine Practitioners. The study explores various metrics and competency frameworks used to assess competency, comparing them against national accreditation schemes' professional capability standards. The findings suggest that accreditation frameworks play a crucial role in improving the quality of paramedicine practice, encompassing intellectual skills, safety, accountability, clinical decision-making, professionalism, communication, teamwork, and situational awareness.

**Supplementary Information:**

The online version contains supplementary material available at 10.1186/s12909-023-04992-w.

## Background

Throughout history, accreditation has been recognised not merely as a mechanism for ensuring the production of safe and competent practitioners and the establishment of minimal educational prerequisites [1]. Recent literature consistently reinforces this perspective [2]. It is imperative to acknowledge that accreditation should not only delineate minimum standards with achievable outcomes but also guarantee perpetual adherence to these educational standards, thus safeguarding the public's welfare. Moreover, accreditation should serve as a catalyst for institutions, compelling them to engage in continuous self-improvement and the evolution of curricula in alignment with the dynamic landscape of practitioner practice [1, 3].

Beyond its advantages for students, higher education, and the general public, accreditation is pivotal in benefiting the respective professions by ensuring the current and future competencies required within practitioners' scopes of practice [4]. Furthermore, accreditation establishes a symbiotic relationship between the profession and educational institutions, standardising curricula and enhancing graduates' preparedness for the workforce [2].

However, the dissenting voices within the discourse on health education accreditation are noteworthy. Critics argue that accreditation may impede curricula' individuality and innovative potential [5]. Additionally, concerns are raised about the associated drawbacks, including escalated costs for institutions, temporal constraints on academic staff, potential student attrition, and the burden of frequently adapting curricula to align with evolving accreditation requirements [6]. While the critics raise valid concerns about the potential drawbacks of health education accreditation, it’s essential to recognise the dynamic nature of healthcare disciplines, exemplified by the field of paramedicine. Paramedicine has traditionally been lauded for its adeptness in delivering early interventional emergency pre-hospital care, thereby contributing significantly to reducing morbidity and mortality [7]. However, the evolving landscape of healthcare demands adaptability, prompting paramedicine to expand its scope into primary healthcare.

In this context, accreditation becomes a pivotal consideration. While accreditation processes are designed to ensure and uphold educational standards, their application to a versatile discipline like paramedicine raises pertinent questions. The traditional framework of accreditation, with its emphasis on standardisation, may pose challenges to the inherent flexibility required in paramedicine's evolving scope. The need for adherence to accreditation requirements might introduce constraints on the innovative approaches that paramedicine often employs in response to emerging healthcare needs.

The changing scope of practice in paramedicine is a result of evidence-based practice, where paramedics deliver care in diverse roles and settings, utilising the latest treatments and technology to serve communities effectively [8, 9]. Paramedics have also seen the profession grow in the diversity of clinical care [10], contributing to managing chronic conditions, acute presentations of mental ill-health, and community health assessments [8]. With paramedicine's evolving scope of practice, education can be at the forefront and a driving force behind these new roles.

As the scope of practice in paramedicine rapidly evolves [10], it is essential to make changes in paramedic education. These changes should not only focus on current practices but also consider future practices [11]. Paramedic university education needs to be updated to include the latest skills, knowledge, and competencies reflective of the evolving scope of practice. In essence, the link between practice, competencies, and education is like a chain reaction. Changes in practice necessitate updated competencies, and to develop those competencies, education needs to adapt. It's a dynamic relationship where each component influences and depends on the others to maintain a high standard of care in an ever-evolving field. Therefore, this paper aims to explore the role of paramedic accreditation in ensuring that the evolving scope of practice in paramedicine is met. To achieve this, we will describe the competencies that graduates must possess and the curriculum that aims to develop these competencies, which are suitable for the evolving scope of practice. To provide a comprehensive understanding, we will undertake a narrative review of the relevant literature.

The narrative review aims to assess whether the professional capabilities in paramedicine align with evidence-based practices and are responsive to the dynamic scope of the field. It will examine the framework set by the national accreditation scheme for paramedicine, with a focus on evaluating the Paramedicine Board of Australia's Professional capabilities for Paramedicine Practitioners. Within the context of this study, Paramedicine Practitioners are defined as paramedics with expanded skills in primary healthcare capabilities. The objective is to determine if these capabilities have been systematically measured and validated in a reliable manner, ensuring they align with current evidence and adapt to the evolving landscape of paramedicine practice.

The PBAs Professional Capabilities are a set of standards developed by the Australian Health Practitioner Regulation Agency (Ahpra) to outline the essential attributes and skills that paramedics need to demonstrate in their professional practice. These capabilities serve as a framework to ensure that practitioners meet the necessary standards of competence and conduct. The development of these professional capabilities often involves a comprehensive process that includes input from experienced paramedics, educators, researchers, and other stakeholders in the field.

In summary, it is contended that the evolving scope of practice necessitates a re-evaluation of the current professional capabilities. The imperative to update these capabilities is underscored by an assertion that accreditation not only ensures the profession's vitality but also enhances the readiness of graduates for the dynamic demands of the workforce. In the context of this discussion, it becomes essential to delineate between competence, competency, and competency frameworks and capabilities for a comprehensive understanding.

## Competence, competency, competency framework and professional capabilities

### Competence

Epstein & Hundert (2002) define competence as the skilled integration of various elements in daily practice for the benefit of individuals and communities served [12]. The distinction between declarative and procedural knowledge is vital—knowing facts does not automatically equate to competence. Declarative knowledge involves understanding concepts [13], while procedural knowledge is about applying skills [14]. Achieving true competence requires a grasp of both types, especially in healthcare education [15]. However, competence extends beyond knowledge; it embodies the attributes of a profession. Simply knowing a subject doesn't guarantee practical competency. It's essential to bridge the gap between knowledge and its application for safe and competent task performance [16].

### Competency

Whiddett's (2003) definition of competency, widely utilized in the development of frameworks, standards, and competency-based education, describes competencies as "behaviors that individuals demonstrate when undertaking job-relevant tasks effectively within a given organizational context" [17]. In the context of traditional higher education, competency tends to be more implicit than explicit [18]. Traditional models assume that completing a degree program implies the acquisition of necessary skills and knowledge. However, these competencies are often not explicitly stated or measured. Unlike traditional models, competency-based education explicitly identifies and measures the specific competencies required by employers or professional organizations, highlighting the need for a more precise assessment of competency levels [18]. The literature now encompasses various higher education health and medical qualifications embracing competency-based education [18–20].

### Competency framework

Ensuring healthcare students graduate as competent professionals is a core objective for higher education institutions. This requires the application of competency frameworks to identify and measure competencies [21]. Accreditation bodies often utilize these frameworks to enhance health programs and students' abilities [21]. However, there's a risk of reducing education to a checklist, prioritizing accreditation over students' application of knowledge in real-world scenarios. It's crucial for institutions to use competency frameworks without compromising the broader goal of developing competent healthcare professionals capable of delivering high-quality care to patients.

### Professional Capabilities

While competencies are reflective of the foundational skills a paramedic requires, and the competency framework is the roadmap in attaining these skills, generally from novice to mastery level. Professional capabilities are identified as the highest level of achievement in meeting competencies [22]. For example, reaching the "Clinical Decision-Making Expert" capability means not just having individual skills but using them effectively in challenging situations.

## Methods

A narrative review was conducted to search for specific competency frameworks for paramedicine and paramedic education. This review builds on the findings from two previous scoping studies that outline competency frameworks for paramedicine and paramedic education [23, 24]. Paramedic education-specific development of standards of practice, competency maps, competency frameworks, competency-based conceptual models and competency assessment tools were selected. It has been previously published that two themes may become evident when defining competency frameworks: the description of work tasks, referred to as competence, and the description of attributes, referred to as competency [17]. Therefore, these terms will be included. This study utilised Arksey & O’Malley’s (2005) methodological framework [25]. A narrative review methodology was applied to focus intentionally, but broadly, on purpose-relevant, theoretically derived research that could inform a competency framework in paramedicine. The relevant articles were classified into two groups: (a) competency assessment tools; and (b) competency frameworks.

EMBASE, EBSCO, Medline, CINAHL, ScienceDirect and PubMed were searched using Boolean operators AND and OR. The search was conducted using the keywords 'paramedic education,' OR 'paramedic higher education,' AND ‘paramedic competence,' and 'paramedic competency frameworks’ and 'paramedic competency’. The search was further refined by a snowball strategy identifying relevant references within each article from the primary search. The initial search was performed on 16 November 2022, and the final search was conducted on 4th December 2023. This study considered peer-reviewed journal articles published between 1994 and 2023. The year 1994 was the year when two universities first introduced paramedic education in the higher education sector in Australia; therefore, it can reasonably be assumed that research and literature around paramedic higher education in Australia and New Zealand commenced after this specified date. The saturation point was reached when no relevant published literature was identified beyond 2023. Secondly, a review of grey literature was undertaken, specifically paramedicine accreditation bodies and professional bodies reports.

**Fig. 1:**
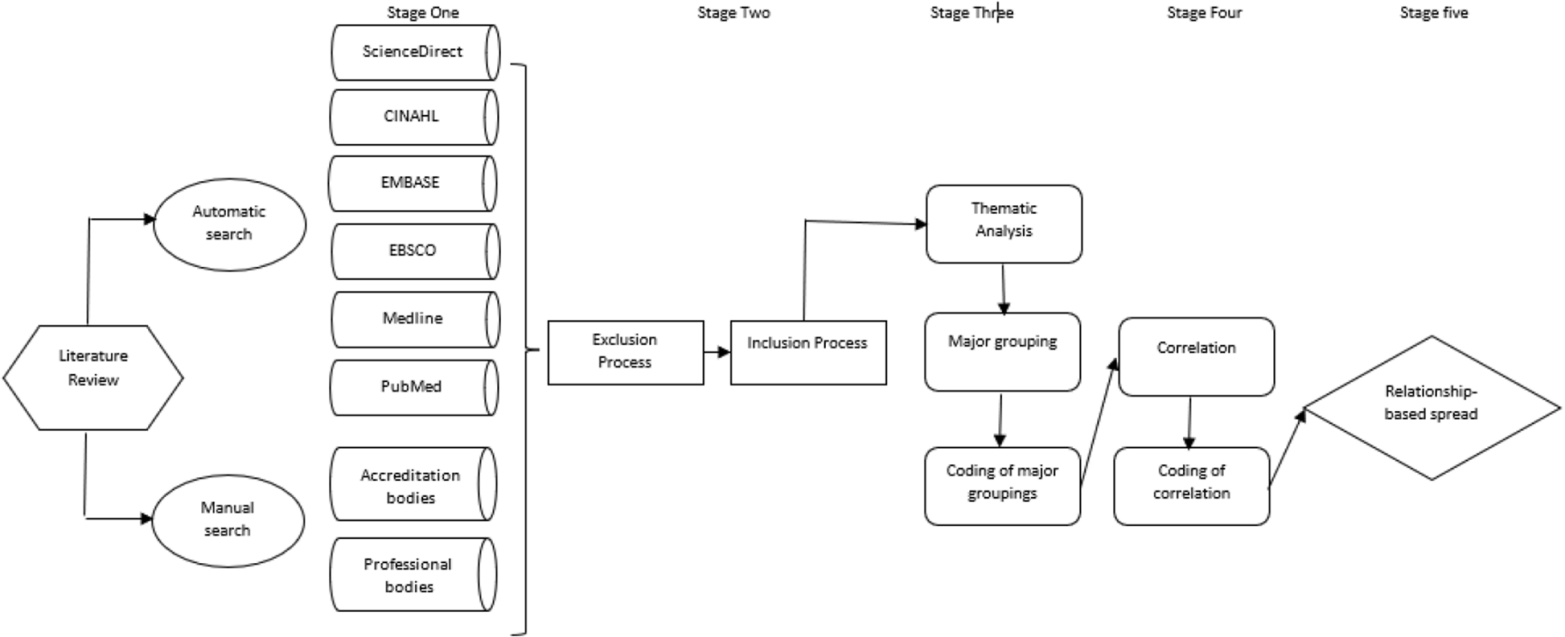
Methods process

The review process involved five stages. Figure 1 outlines the process, and the steps undertaken to analyse the quantitative data to determine a relationship between the findings of the literature review against the PBA Professional Capabilities. The process commenced with a search of peer-reviewed journal articles in several databases. We then manually searched paramedicine accreditation bodies' standards and professional bodies' reports. We then excluded articles that were not related to the search terms. The inclusion process included filtering articles based on abstract or where the abstract was identified as vague; the introduction was then reviewed. Stage three involved identifying themes relevant to a competency framework. These themes were then grouped into major headings and coded in Microsoft excel. Stage four involved identifying a relationship between the significant groupings and the five domains for the professional capabilities of registered paramedics. Text network analysis as the core framework for text mining and textual data analysis was undertaken identifying the significant groupings against the domains involved a content analysis of the domains and matching words or similar words from the major groups (e.g., communication, communication skills, interpersonal communication). Finally, bubble charts display three data dimensions using a relationship-based visual spread resembling a scatter plot. Entities—represented as spheres—are plotted on the chart's axes according to two variables and sized according to the third variable. The first variable was the findings from the literature review that were put into themes, the second variable was the mapping of these themes to PBA's professional capabilities, and thirdly, the bubble size reflected the relationship between the findings in the literature to the professional capabilities.

## Results

The final inclusion process yielded 62 articles; all except one met the final inclusion process. Fifteen articles were grey literature, and one dissertation was also reviewed.. Due to the exploratory nature of this study, the articles were categorised into two groups, (a) competency assessment tools; and (b) competency frameworks.

The thematic analysis approach proposed by Braun and Clarke (2017) involves a systematic process of identifying, analysing, and reporting patterns, or themes, within qualitative data. At its core, thematic analysis serves as a robust foundation for qualitative research [26]. For the thematic analysis, we employed a hybrid approach that incorporated both inductive and deductive elements. Initially, the coding process began with open coding, allowing for the emergence of codes directly from the data in an inductive manner. Subsequently, these codes were iteratively organized into broader categories based on existing literature and theoretical frameworks—a deductive process. This hybrid approach provided a nuanced understanding of the data while ensuring alignment with established concepts in the field.

In the initial stage of our analysis, we identified a substantial dataset comprising 29 competency frameworks and 249 core competencies. These 278 themes were initially treated as descriptive, aligning with the approach of Thomas and Harden (2008), who emphasised the importance of translating descriptive themes into analytical ones for a more nuanced interpretation and hypothesis generation [27].

In our review process, we meticulously organised the data into major groupings, recognising the inherent complexity in grouping concepts and themes [26]. The synthesis unfolded through three distinct stages: firstly, a detailed, free line-by-line coding of the literature review findings; secondly, the organisation of these 'free codes' into related areas to construct 'descriptive' themes; and finally, the development of major groupings that encapsulated and distilled the essence of the 278 themes into 22 significant analytical categories (see Table 1).

**Table 1:** Major analytical groupings

Accountability	Assessment	Clinician (effective clinical care)	Clinical skills
Clinical decision making (CDM)	Cultural competency	Communication	Educator
Health and Social continuum	Information literacy	Intellectual skills	Leadership
Learning and professional development	Mentoring and clinical supervision	Professionalism	Research
Safety	Scene management	Self-directed practice	Situation awareness
Team approach		Be able to work in different transportation modes.	

We took a quantitative approach to examine how often themes associated with connection appeared in the dataset. Themes were coded based on their connection relevance, and we tallied the frequency of each theme. Assessing the distribution of these themes across the dataset, considering both prevalence and significance, helped determine the degree of association. This method enabled us to quantitatively describe the strength and prominence of connections within the analysed data.

**Fig. 2:**
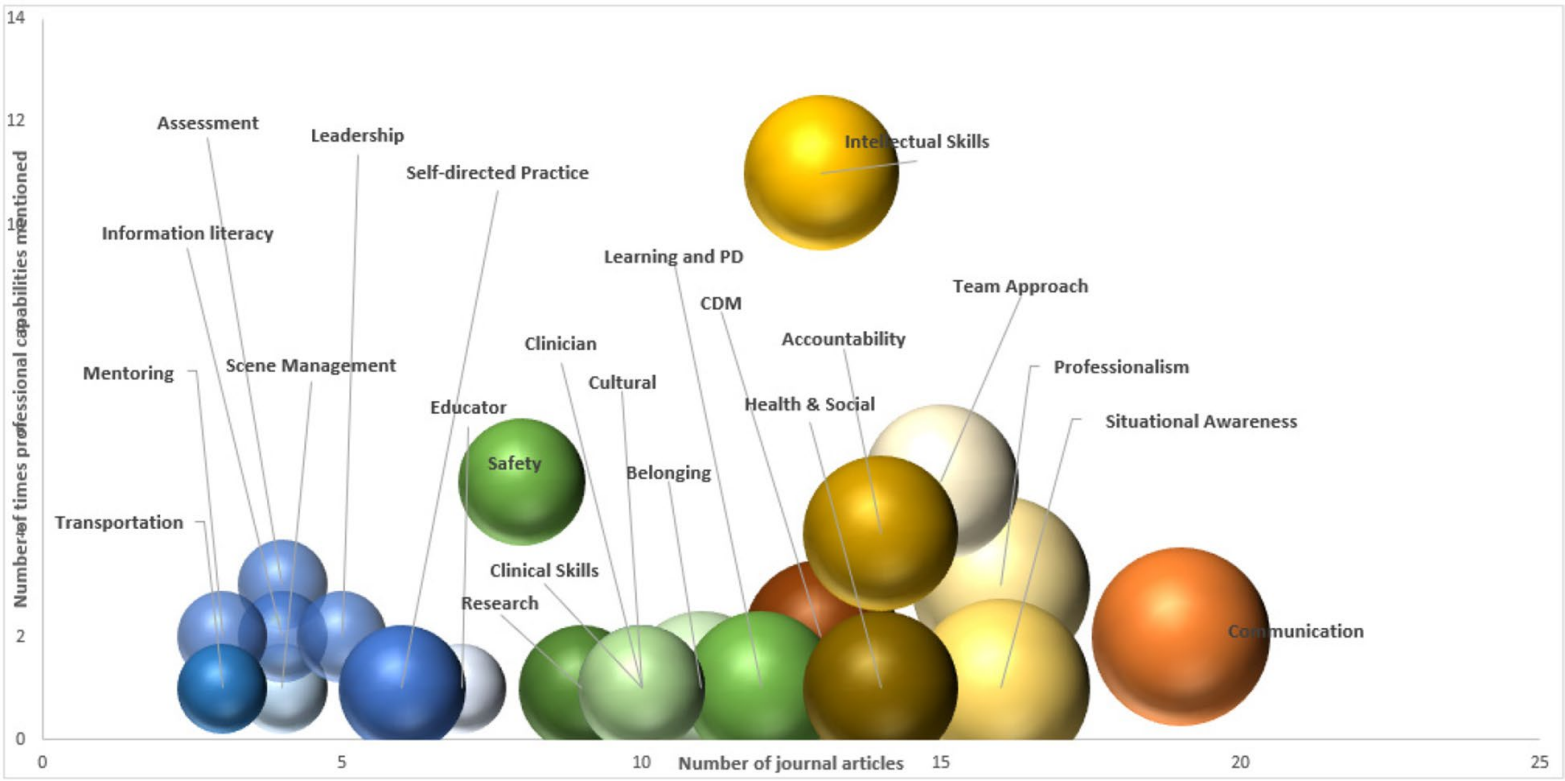
Relationship between findings from the literature review against the PBA Professional Capabilities

**Fig. 3:**
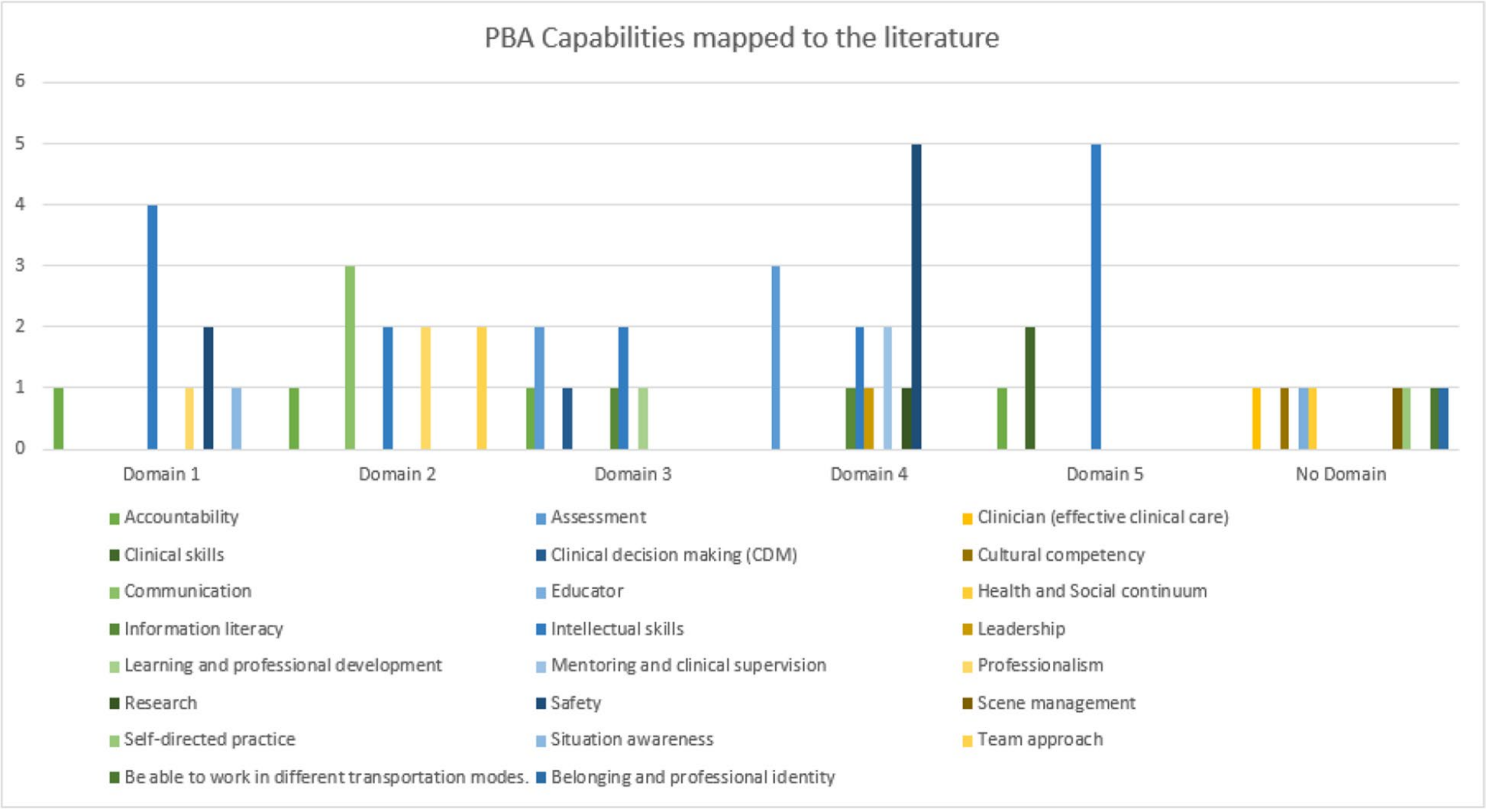
PBA Professional Capabilities mapped to the findings in the literature

Visual representation of data, such as scatter plots and bubble charts, has previously been recommended for qualitative data display to present and summarise findings ([28]. To provide a better representation of the relationship between the number of times the major analytical groupings were represented in the literature, the depiction of the groups as identified in the domains of the PBA professional capability and the relationship of these groupings to the PBA professional capability, a visual representation of the data is presented [28]. Figure 2 depicts a mapping of the number of documents in the major grouping highlighted in the literature, as shown on the *Y* axis. The number of times the major grouping was identified within one or more domains of the PBA professional capability is shown on the *x*-axis. The size of the bubble represents the relationship between the two. Figure 3 represents mapping of the PBA Capabilities against the findings in the literature.

We identified that intellectual skills were the most prevalent group within the literature, identified within the domains, and posed an excellent association. Intellectual skills were embodied within the literature to demonstrate the knowledge required to practice as a paramedic. They included terms such as knowledge base, educational background, theoretical, and course-based knowledge. Safety was the second grouping identified to be prevalent within two or more domains of professional capabilities; however, it was lower than intellectual skills within the literature. The result is a medium relationship between the literature and the PBA identified within the domains.

The data analysis highlighted accountability, clinical decision-making, professionalism, communications, team-based approach and situational awareness were the highest-rated groupings within the literature and were identified within one or more domains. The result strongly correlates with the literature findings and the PBA professional capabilities.

On average, providing effective clinical care, clinical skills, cultural competency, learning and professional development and research were identified within ten articles. However, these groupings were moderately represented within the domains, averaging only two mentions.

Finally, it was revealed within the analysis that the lowest-ranked groupings were assessment, education, Health and Social continuum, information literacy, leadership, mentoring, scene management and self-directed practice. These groupings were mentioned within five or fewer articles and up to four times in the domains. The lowest-ranked groupings were Health and Social continuum and self-directed practice, represented in five articles but should be mentioned within the PBA professional capabilities. These factors have previously been identified as dimensions of professional competence and have been further categorised into cognitive, technical, integrative, context, relationship, affective/moral and habits of mind [12].

## Discussion

The paper aimed to systematically examine existing literature on paramedic evolving scope of practice, aligning these insights with the PBA’s professional capabilities. The objective was to assess the evidence-based validity of the professional capabilities and identify potential gaps in the current framework.. The results of this narrative review of the literature and mapping study indicate various metrics of what the literature perceives as (a) competency assessment tools; and (b) competency frameworks against the national accreditation schemes' professional capability.

The findings from our literature review and mapping study shed light on various metrics related to competency assessment tools and competency frameworks in comparison to national accreditation schemes' professional capabilities. Accreditation plays a crucial role in ensuring the quality of educational programs. However, the need for more evidence on the assurance of quality achieved through accreditation, as highlighted by Phillips and Kinser (2018),raises important considerations [29]. There needs to be more evidence as to whether an assurance of quality is achieved through accreditation [30].

A framework has previously been defined as the foundational structure supported by facts, concepts, theories, and propositions [31, 32]. It is a term recognisable in the educational sector as necessary due to the independent review of learning activities [32]. A framework develops clear, high-quality standards , with findings from one Delphi study identified that accreditation is crucial for quality improvement [33]. More recently, a report by the World Health Organization described accreditation frameworks as quality interventions that prepare healthcare institutions before and during the accreditation process [33]. These definitions have a common theme: a framework for accreditation is a foundation for quality improvement.

Expanding on the definitions of accreditation and frameworks, our study identified that an accreditation framework encompasses various elements, including intellectual skills, safety, accountability, clinical decision-making, professionalism, communication, team-based approach, and situational awareness. These elements form the basis for being an effective clinician, applying clinical skills, and engaging in ongoing professional development.

Transitioning to our specific findings, the data were categorised into major groupings through a rigorous three-stage synthesis process. Intellectual skills emerged as the most prevalent group, demonstrating a strong relationship with the knowledge required for paramedic practice. Safety, while prevalent, showed a medium relationship with the PBA professional capabilities.

Further analysis highlighted that accountability, clinical decision-making, professionalism, communication, team-based approach, and situational awareness were the highest-rated groupings in the literature, aligning strongly with both the literature findings and the PBA professional capabilities.

On the other hand, groupings such as effective clinical care, clinical skills, cultural competency, learning, and professional development were moderately represented within the domains. Finally, the analysis revealed that the lowest-ranked groupings, including assessment, education, Health and Social continuum, information literacy, leadership, mentoring, scene management, and self-directed practice, were mentioned in fewer articles and fewer times within the domains. Notably, Health and Social continuum and self-directed practice, though represented in five articles, should be emphasised within the PBA professional capabilities, as they contribute to dimensions of professional competence.

### Intellectual skills

The discussion on cognitive and technical competence is crucial in the context of paramedicine, as it forms the backbone of effective paramedic care. The emphasis on evidence-based paramedical care underscores the need for a strong foundation in intellectual skills. This includes a comprehensive knowledge base, educational background, theoretical understanding, and course-based knowledge—essential components that contribute to the competency framework for paramedics [23].

As the field of paramedic clinical care evolves, it becomes evident from the literature that staying abreast of changes in medicine and pre-hospital care is paramount [33–36]. The shift towards higher education in paramedic training is supported by the literature, emphasising the interrelationship between knowledge and practice in preparing students to be job-ready [37]. The acknowledgement that higher-order critical thinking and problem-solving skills, backed by evidence-based practice, are best cultivated through degreed paramedics, aligns with the changing landscape of paramedic education [38].

The study's findings shed light on the prevalence of intellectual skills as a cornerstone of paramedic competence, with a medium relationship to the identified Professional Capability Domains. Safety, while significant, ranks lower than intellectual skills, suggesting a nuanced interplay between theoretical knowledge and practical application.

The discussion on cognitive and technical competence in paramedicine not only underscores the importance of intellectual skills but also emphasises the need for a holistic approach that integrates various dimensions of professional competence. The evolving nature of paramedic clinical care necessitates a dynamic and adaptive education system that equips paramedics with the diverse skills required for optimal patient care.

### Accountability, clinical decision-making, professionalism, communication, team-based approach, and situational awareness

The scope of practice and educational requirements for paramedics have transformed significantly over the past four decades, with the COVID-19 pandemic further emphasising the shifting role of paramedicine within the healthcare continuum [39]. Paramedics are now presented with opportunities beyond emergency responsiveness, expanding their roles into chronic and specialist services in primary care, preventive measures, and public health. The increased scope of practice requires paramedics to make complex, cost-effective clinical judgments about patient management and transport to hospitals, reflecting the need for a broader skill set and knowledge base [10].

The findings highlight the critical importance of relationships in paramedicine, emphasising the positive impact on patient outcomes when paramedics build trust, respect, empathy, and effective communication with patients, colleagues, and interdisciplinary teams [40, 41]. Communication skills, grounded in general communication strategies and psychological foundations, are deemed crucial for enhancing paramedic competency [42].

The affective/moral dimension addresses the preparedness of paramedic students to practice, emphasising the role of communication and professionalism skills in ensuring road readiness and minimising deficits for employers [43]. Collaboration between higher education institutions and employers is identified as vital for balancing work requirements and learner experience [44, 45]. The habits of mind in paramedicine, encompassing self-reflection, self-monitoring, and awareness of one's mental, emotional, and physical abilities, are crucial for professional development [46]. However, the study notes a lack of attention to these aspects in both practice and education.

Despite the identification of many competency assessment tools and frameworks, the study reveals a gap in correlating Health and Social continuum and self-directed practice, cornerstone competencies for primary healthcare [46]. Paramedics, as essential resources in health prevention, are positioned beyond emergency care, integrated with health, aged care, and social services [12, 47].

The association between the study's findings and the Professional Capability Domains (accountability, clinical decision-making, professionalism, communications, team-based approach, and situational awareness) supports the literature's emphasis on these areas. However, certain crucial aspects, including providing effective clinical care, clinical skills, cultural competency, learning, professional development, and research, are moderately represented within the domains, signalling a need for further attention and integration into paramedic education.

### Clinical care, clinical skills, cultural competency, learning, and professional development

The results of the study highlight a noteworthy discrepancy in the representation of certain crucial areas within the paramedicine profession. Specifically, the dimensions of providing effective clinical care, clinical skills, cultural competency, learning and professional development, and research emerged as focal points in the literature, with an average of ten mentions across various sources. However, the moderation of their representation within the identified domains, averaging only two mentions, brings forth important considerations for the future development and refinement of paramedic education and practice.

The prominence of providing effective clinical care and clinical skills in the literature underscores their foundational significance in paramedicine. These aspects form the bedrock of paramedic competency, directly impacting patient outcomes and the overall quality of pre-hospital care. However, the moderate representation within the domains suggests a potential gap in the integration of these critical components into the recognised Professional Capability Domains.

Cultural competency, identified as a key dimension in the literature, reflects the growing recognition of the diverse communities paramedics serve. Understanding and respecting cultural nuances are vital for delivering patient-centred care. The moderate representation within the domains calls attention to the need for a more robust integration of cultural competency within the recognized professional capabilities, ensuring that paramedics are equipped to navigate the diverse cultural landscape they encounter in their practice.

Learning and professional development emerge as pivotal dimensions, acknowledging the dynamic nature of healthcare and the continuous evolution of paramedic roles. The literature emphasizes the importance of ongoing education to keep pace with the changing demands of the profession. The moderate representation within the domains suggests a potential gap in acknowledging and structurally embedding continuous learning and professional development as integral components of paramedic competency.

Research, another dimension highlighted in the literature, underscores the importance of evidence-based practice in paramedicine. The ability to critically evaluate and apply current research findings is crucial for ensuring the delivery of optimal patient care. The moderate representation within the domains signals an opportunity to strengthen the emphasis on research skills as an inherent part of paramedic competency.

Addressing these discrepancies requires a thoughtful and comprehensive approach to curriculum development, accreditation frameworks, and ongoing professional training. Integrating these dimensions more prominently within the recognized domains ensures that paramedics receive a well-rounded education that aligns with the diverse and dynamic demands of their profession. It is essential for accrediting bodies, educators, and practitioners to collaborate in refining the competency framework to encompass these critical dimensions, thereby enhancing the overall competence and preparedness of paramedics in the ever-evolving healthcare landscape.

The literature underscores the importance of establishing a conceptual framework as the foundation for paramedicine education, shaping educational practices related to a paramedic's modus operandi (ontology), declarative knowledge (epistemology), clinical judgment (ethics), and procedural knowledge (praxis) [38, 48]. Various papers have highlighted the need for more literature in this area. The absence of frameworks in paramedicine education and accreditation necessitates the creation of a comprehensive framework specifically designed to support paramedic clinical judgment and decision-making skills [49, 50].

One such framework, the complex adaptive system, emerges as a viable option to guide paramedicine's role in the primary healthcare sector [51]. Positioned within the paramedic's roles and responsibilities, this framework signifies a diverse and dynamic working environment that demands rapid adaptation and ongoing quality enhancement, all while being interconnected with other healthcare services [39]. Recommending the use of this framework as the domains for professional capabilities by accreditation bodies could provide a structured and cohesive approach to paramedic education and accreditation, ensuring alignment with the complex and evolving nature of the paramedic profession.

## Conclusion

In conclusion, our examination of the existing literature on the evolving scope of practice in paramedicine serves as a crucial foundation for aligning insights with the PBA's professional capabilities. The primary objective was to rigorously assess the evidence-based validity of these capabilities and concurrently identify potential gaps within the current framework.

Our approach, grounded in a comprehensive literature narrative review and guided by Arksey & O’Malley’s methodological framework, aimed at providing a nuanced understanding of the evolving landscape. This was further complemented by a robust thematic analysis, employing both inductive and deductive elements, to uncover 278 themes distilled into 22 analytical categories.

The visual representation of data through tables and bubble charts facilitated a deeper comprehension of the relationships between major analytical groupings and the PBA professional capabilities. This methodological transparency not only enhances the credibility of our conclusions but also enables readers to identify the gaps in the professional capabilities against the evolving scope of practice of paramedicine.

Our findings underscored the significance of key analytical groupings, highlighting areas of strength such as accountability, clinical decision-making, professionalism, communication, team-based approach, and situational awareness. Conversely, lower-ranked groupings identified potential areas for attention and further exploration.

In essence, this systematic exploration has not only contributed to the understanding of the evolving scope of practice in paramedicine but has also shed light on the alignment of these insights with the PBA's professional capabilities. The evidence-based approach employed throughout our study not only validates the current framework but also opens avenues for future refinement and development practice.

### Supplementary Information

**Additional file 1: Appendix 1.**

## Data Availability

The datasets used and/or analyzed during the current study available from the corresponding author on reasonable request.
