# Supplementary material for: Exploring the alignment between paramedicine's professional capabilities and competency frameworks for current and evolving scopes of practice: a literature review
Source: BMC Med Educ. 2024 Jan 5;24:31. doi: 10.1186/s12909-023-04992-w (PMC10768442; doi:10.1186/s12909-023-04992-w)
Supplement: Supplementary file 1 — Additional file 1: Appendix 1. [file 12909_2023_4992_MOESM1_ESM.doc]

Appendix One

| Citation | Article type | Notes |
| --- | --- | --- |
| Tavares, W., Boet, S., Theriault, R., Mallette, T., & Eva, K. W. (2012). Global Rating Scale for the Assessment of Paramedic Clinical Competence. Prehospital Emergency Care, 17(1), 57–67. https://doi.org/10.3109/10903127.2012.702194 | modified Delphi process | The aim of this study was to develop and critically appraise a global rating scale (GRS) for the assessment of individual paramedic clinical competence at the entry-to-practice level. |
| Tavares, W., & Boet, S. (2016). On the Assessment of Paramedic Competence: A Narrative Review with Practice Implications. Prehospital and Disaster Medicine, 31(1), 64–73. https://doi.org/10.1017/S1049023X15005166 | Narrative Review | The assessment of paramedic competence is a complex process requiring an understanding, appreciation for, and integration of conceptual and psychometric principles |
| O’Meara, P. F., Furness, S., & Gleeson, R. (2017). EDUCATING PARAMEDICS FOR THE FUTURE: A HOLISTIC APPROACH. Journal of Health and Human Services Administration, 40(2), 219–253. | Case Study | PARiHS conceptual framework- Promoting Action on Research Implementation in Health Sciences (PARiHS) Framework, was used to address the complexity of developing and implementing an evidence based paramedicine curriculum |
| McCallum, M., Carver, J., Dupere, D., Ganong, S., Henderson, J. D., McKim, A., McNeil-Campbell, L., Richardson, H., Simpson, J., Tschupruk, C., & Jewers, H. (2018). Developing a Palliative Care Competency Framework for Health Professionals and Volunteers: The Nova Scotian Experience. Journal of Palliative Medicine, 21(7), 947–955. https://doi.org/10.1089/jpm.2017.0655 | literature search | Nova Scotia Palliative Care Competency Framework. |
| Six steps in the right direction: guiding the development of competency frameworks in healthcare professions. 1. Alan M. Batt MSc PhD(c) 2. Brett Williams PhD 3. Madison Brydges MA PhD(c) 4. Matthew S. Leyenaar MA PhD(c) 5. Walter Tavares PhD | Concept development | Improving Competency Framework Development: A Six-Step Model |
| Petter, J., & Armitage, E. (2012). Raising educational standards for the paramedic profession. Journal of Paramedic Practice : the Clinical Monthly for Emergency Care Professionals, 4(4), 241–242. https://doi.org/10.12968/jpar.2012.4.4.241 | Discussion Paper | The College of paramedics influence on professional standards- College’s Curriculum Guidance and Competency Framework |
| Cooper SContemporary UK paramedical training and education. How do we train? How should we educate?Emergency Medicine Journal 2005;22:375-379. | qualitative naturalistic inquiry using an interpretative constructivist approach | clear need to identify alternative methods of assessment such as self and peer review and objective structured clinical examinations with links to a competency framework. |
| Tavares, W., Bowles, R., & Donelon, B. (2016). Informing a Canadian paramedic profile: framing concepts, roles and crosscutting themes. BMC Health Services Research, 16(1), 477–477. https://doi.org/10.1186/s12913-016-1739-1 | Discussion paper | For Canada, the Paramedic Association of Canada’s (PAC) National Occupational Competency Profile (NOCP) defines Canadian paramedic practice nationally by describing the competencies or tasks paramedics are expected to possess when practicing at a specified level |
| Donaghy, J. (2010). Equipping the student for workplace changes in paramedic education. Journal of Paramedic Practice : the Clinical Monthly for Emergency Care Professionals, 2(11), 524–528. https://doi.org/10.12968/jpar.2010.2.11.80045 | grounded theory | key areas of the curriculum, in addition to student familiarization with equipment, both of which students are expected to have a degree of knowledge and competency prior to attending practice, and ultimately meet the entry level requirement of Standards of Proficiency for eligibility for registration with the Health Professions Council (HPC 2006). |
| Paramedics Australasia 2011 | Grey literature | Australasian Competency Standards for Paramedics |
| Assessing the Practice Competence of Undergraduate Students in Paramedicine in Australia and New Zealand: A Grounded Theory Study | Grounded Theory | Dissertation |
| Weber, A., Lawson, C., & Williams, B. (2021). Frameworks that guide curriculum development in Australian higher education. Journal of Paramedic Practice : the Clinical Monthly for Emergency Care Professionals, 13(3), 105–112. https://doi.org/10.12968/jpar.2021.13.3.105 | focused discourse analysis of the grey literature | Three of the 18 institutions in Australia and New Zealand that offer paramedic education identified some form of framework. Two theories were identified as applicable in supporting the development of curricula that are focused on the expanded scope of practice of paramedicine. |
| AlShammari, T., Jennings, P., & Williams, B. (2019). Emergency medical services core competencies: a Delphi study. Australasian Journal of Paramedicine, 16. https://doi.org/10.33151/ajp.16.688 | Delphi study | This study provided consensus on 41 core competencies specific to Saudi EMS industry requirements. However, the findings do not represent a definitive blueprint model for alignment into EMS curricula. |
| College of Paramedics, 2019 | Grey Literature | Macmillan AHP competency framework which has been endorsed by the College of Paramedics is aligned to UKONS Acute Oncology Competence Framework |
| College of Paramedics | Grey Literature | Paramedic Curriculum Guidance |
| South Yorkshire Workforce and Training Hub | Grey Literature | Role Description & Competencies: Paramedic |
| Tackett S, Sugarman J, Ng CJ, et alDeveloping a competency framework for health research ethics education and trainingJournal of Medical Ethics 2022;48:391-396. | Framework development | We detail critical questions for developing HRE competency frameworks using a six-step process: (1) define the purposes, intended uses and scope of the framework; (2) describe the context in which practice occurs; (3) gather data using a variety of methods to inform the competency framework; (4) translate the data into competencies that can be used in educational programmes; (5) report on the competency development process and results and (6) evaluate and update the competency framework. |
| Reid, D., Street, K., Beatty, S., Vencatachellum, S., & Mills, B. (2019). Preparedness of graduate paramedics for practice: a comparison of Australian and United Kingdom education pathways. Australasian Journal of Paramedicine, 16. https://doi.org/10.33151/ajp.16.666 | semi-structured face-to-face interviews | jurisdiction defines their individual professional paramedic competencies or professional capabilities that must be met by graduate paramedics. In Australia, competencies/standards of proficiency are set by the CAA and are now set by AHPRA, and in the UK they are set by the HCPC |
| Te Kaunihera Manapou Paramedicine Board, NZ 2020 | Grey Literature | Standards of Clinical Competence for Paramedics |
| AlShammari, T., Jennings, P., & Williams, B. (2019). National study of emergency medical services core competencies: a confirmatory factor analysis. Australasian Journal of Paramedicine, 16. https://doi.org/10.33151/ajp.16.706 | confirmatory factor analysis | utilise confirmatory factor analysis (CFA) through structural equation modelling to confirm the theoretically developed Saudi ParamEdic Competency Scale (SPECS) model. |
| Jennifer Bolster1-3, Priya Pithia1, and Alan M. Batt - Emerging Concepts in the Paramedicine Literature to Inform the Revision of a pan-Canadian Competency Framework for Paramedics: A Restricted Review 2022 | A Restricted Review |  |
| 2022 Workforce Development Trust | Grey Literature | Paramedic Specialist in Primary and Urgent Care (2019)- The Paramedic Specialist Core Capabilities Framework comprises the following capabilities, grouped into four domains: Domain A. Person-Centred collaborative working Capability 1. Communication Capability 2. Person-Centred care Capability 3. Working with families and carers Capability 4. Referrals and integrated working Capability 5. Law, ethics and safeguarding Domain B. Investigation, assessment, advice and clinical impression or diagnosis Capability 6. History-taking and consultation skills Capability 7. Physical and mental health assessment Capability 8. Investigations and diagnosis Domain C. Condition management, treatment and prevention Capability 9. Treatment and care planning Capability 10. Pharmacotherapy Capability 11. Health promotion and lifestyle interventions Domain D. Leadership and management, education and research Capability 12. Leadership and management Capability 13. Education Capability 14. Research |
| BC Emergency Health Services, 2022 | Grey Literature | Competencies To be successful, you will require the following skills and abilities: Self-awareness and self-management Take responsibility for your own performance and health The qualities of reliability, honesty, integrity, resilience and confidence Actively seek opportunities and challenges for personal learning, character building and growth and take action to achieve your goals Willingness to work through the personal experience of an unsuccessful outcome and be able to learn from it Keep your emotions under control when provoked, faced with opposition or hostility from others, or when working under stress. Communicate intentions, ideas and feelings openly and directly, and welcome openness and honesty even in difficult negotiations Thinking skills Systematically organize and compare aspects of a problem and determine “if this is done then that will happen” Identify patterns or connections between situations that are not obviously related and categorized by linear thinking Make the best decisions possible with the information available Engagement and awareness of others Adapt and connect with others to work effectively in a variety of diverse situations with diverse individuals or groups Work collaboratively by valuing the diversity of others and fostering respect and equity in the workplace Encourage team members to cooperate, support each other, share knowledge and expertise Listen well and encourage the open exchange of information and ideas |
| Health Education England- First Contact Practitioners and Advanced Practitioners in Primary Care: (Paramedic) A Roadmap to Practice, 2021 | Grey Literature | This document provides a roadmap of education for practice when moving into First Contact Practitioner (FCP) roles, and onward to Advanced Practice (AP) roles in Primary Care |
| College of Paramedics, 2018 | Grey Literature | Paramedic Specialist in Primary and Urgent Care Core Capabilities Framework |
| Eaton, G., Wong, G., Tierney, S., Roberts, N., Williams, V., & Mahtani, K. R. (2021). Understanding the role of the paramedic in primary care: a realist review. BMC Medicine, 19(1), 1–145. https://doi.org/10.1186/s12916-021-02019-z | a realist review | This review is the first to offer insight into understanding the impact paramedics may have on the international primary care workforce and shaping how they might be optimally deployed. |
| Hodge A, Swift S, Wilson JP. Maintaining competency: a qualitative study of clinical supervision and mentorship as a framework for specialist paramedics. British Paramedic Journal. 2018 Dec;3(3):10-15. DOI: 10.29045/14784726.2018.12.3.3.10. | Thematic analysis | The aim of this study was to explore the factors influencing the maintenance of clinical competence and the effectiveness of the specialist paramedic in the context of mentorship, from the specialist paramedic's own perspective. |
| AlShammari, T., Jennings, P., & Williams, B. (2019). National study of emergency medical services core competencies: An exploratory factor analysis. International Journal of Emergency Services Vol. 9 No. 3, 2020 pp. 245-255 | An exploratory factor analysis | The EFA model Saudi ParamEdic Competency Scale (SPECS) has been identified, with 27 core competency items and five overarching factors. |
| Tavares, W., Bowles, R., & Donelon, B. (2016). Informing a Canadian paramedic profile: framing concepts, roles and crosscutting themes. BMC Health Services Research, 16(1), 477–477. https://doi.org/10.1186/s12913-016-1739-1 | concurrent mixed methods study design involving a focused discourse analysis | The paramedic profession is experiencing a shift that appears to deviate or at least place a tension on traditional views or models of practice. Underlying and evolving notions of practice are resulting in intended or actual clinical and professional boundaries that may require the profession to re-think how it is defined and/or shaped. Until these framing concepts, roles and crosscutting themes are fully understood, tested and operationalized, tensions between guiding frameworks and actual or intended practice may persist. |
| The Royal Pharmaceutical Society 2019 | Grey Literature | A Competency Framework for Designated Prescribing Practitioners |
| Richard Brightwell, Bill Lord, Moira Sim, Brian Maguire and Lisa Holmes- Standards and quality indicators for best practice in paramedic and inter-professional experiential practica, 2015 | Grey Literature | experiential clinical learning framework (adapted from (Herrington ... paramedic competencies |
| Talal AlShammari, Paul A. Jennings, Brett Williams, Emergency Medical Services Core Competencies: A Scoping Review, Health Professions Education,Volume 4, Issue 4, 2018, Pages 245-258, | scoping review | The review will provide insight into the scope of knowledge, abilities, skills and education that can be important to the conduct of paramedic students. Moreover, the review would be part of a greater project to develop a set of core competencies specifically designed for Bachelor EMS degrees in Saudi Arabia. This is the first scoping review that has attempted to systematically identify potential core competencies for paramedic students. |
| Batt, A. M., Tavares, W., & Williams, B. (2020). The development of competency frameworks in healthcare professions: a scoping review. Advances in Health Sciences Education : Theory and Practice, 25(4), 913–987. https://doi.org/10.1007/s10459-019-09946-w | scoping review | Competency frameworks serve various roles including outlining characteristics of a competent workforce, facilitating mobility, and analysing or assessing expertise. |
| Tavares, W., & Boet, S. (2016). On the Assessment of Paramedic Competence: A Narrative Review with Practice Implications. Prehospital and Disaster Medicine, 31(1), 64-73. doi:10.1017/S1049023X15005166 | Narrative review | The assessment of paramedic competence is a complex process requiring an understanding, appreciation for, and integration of conceptual and psychometric principles. The field of PBA is advancing rapidly with numerous opportunities for research. |
| Shannon, B., Eaton, G., Lanos, C., Leyenaar, M., Nolan, M., Bowles, K.-A., Williams, B., O’Meara, P., Wingrove, G., Heffern, J. D., & Batt, A. (2022). The development of community paramedicine; a restricted review. Health & Social Care in the Community. https://doi.org/10.1111/hsc.13985 | a restricted review | Explore and better understand the successes and learnings of community paramedic programmes across five domains being; education requirements, models of delivery, clinical gov-ernance and supervision, scope of roles and outcomes |
| Tavares, W., Allana, A., Beaune, L., Weiss, D., & Blanchard, I. (2021). Principles to Guide the Future of Paramedicine in Canada. Prehospital Emergency Care, 26(5), 728–738. https://doi.org/10.1080/10903127.2021.1965680 | in-depth one-onone semi-structured interviews | Conclusions: Our results provide a conceptual framework made up of guiding principles and enablers that provide a consolidated lens to advance the paramedicine profession in Canada (and elsewhere as appropriate) while ensuring contextual and regional needs and differences can be accounted for |
| Canadian Patient Safety Institute, 2015 | Grey literature | Paramedic’s educational framework for patient safety - The Paramedic Association of Canada has since adopted the CanMEDS framework, a competency-based approach to medical education, to align their work and how they will define their profession. |
| Baranowski, L., & Armour, R. (2020). Do non-rotational ambulance-based placements affect the development of paramedic competencies from a student perspective? A qualitative study. Australasian Journal of Paramedicine, 17. https://doi.org/10.33151/ajp.17.703 | semi-structured group interviews | This research provides an initial insight into how paramedic students perceive the effects of non-rotational ambulance-based placements on the development of competencies |
| Prescher, T., Konig, H., & Wiesner, C. (2021). A pedagogical framework for a paradigm shift in emergency paramedic training: Rescue service schools between requirement conflicts and learning culture development. In W. B. James, C. Cobanoglu, & M. Cavusoglu (Eds.), Advances in global education and research (Vol. 4, pp. 1–13). USF M3 Publishing. https://www.doi.org/10.5038/9781955833042 | book chapter | The discussion around changing the culture of teaching and learning with a stronger focus on competence-oriented teaching within a framework of self-development and self-appropriation is not a new discussion in pedagogy |
| TOWNSEND, Ruth M. What Australian and Irish paramedic registrants can learn from the UK: lessons in developing professionalism.. Irish Journal of Paramedicine, [S.l.], v. 2, n. 2, dec. 2017. ISSN 2009-938X. | Discussion paper | To act with professionalism is to act within a set of moral norms, principles and standards of conduct and competency |
| Cooper, S. (2005). Contemporary UK paramedical training and education. How do we train? How should we educate? Emergency Medicine Journal : EMJ, 22(5), 375–379. https://doi.org/10.1136/emj.2004.019208 | qualitative naturalistic inquiry | To develop an understanding of the current system and future development of training and education within a large UK ambulance trust, based upon the experiences, beliefs, and opinions of stakeholders |
| Steve Raven, Vivienne Tippett, Jon-Grant Ferguson, Somma Smith- An exploration of expanded paramedic healthcare roles for Queensland | Grey Literature | monograph publication, which explores expanded paramedic healthcare roles and alternative models of health service delivery. |
| Belinda Flanagan, Matthew Warren-James & Jeanine Young (2022) Evaluation of the CARES Skills Framework as a Peer Support Model in the Paramedic Undergraduate Curriculum: Facilitating Challenging Discussions in a Safe Environment, Prehospital Emergency Care, DOI: 10.1080/10903127.2022.2125136 | modified nominal group technique method | This study evaluated the CARES skills framework (Connect to emotion, Attention training, Reflective listening, Empathy, Support help seeking) as a peer support model to encourage student paramedics to talk about grief and death related to infants and children. |
| Helge Regener (2005) A proposal for student assessment in paramedic education, Medical Teacher, 27:3, 234-241, DOI: 10.1080/01421590500126262 | Concept paper | This paper develops a concept of assessment for paramedic education. |
| Studnek, J. R., Fernandez, A. R., Shimberg, B., Garifo, M., & Correll, M. (2011). The Association Between Emergency Medical Services Field Performance Assessed by High‐fidelity Simulation and the Cognitive Knowledge of Practicing Paramedics. Academic Emergency Medicine, 18(11), 1177–1185. https://doi.org/10.1111/j.1553-2712.2011.01208.x | Observational study | The objective of this study was to assess the association between the performance of practicing paramedics on a validated cognitive exam and their field performance, assessed on a simulated emergency medical services (EMS) response. |
| Lurie, S. J., Mooney, C. J., & Lyness, J. M. (2009). Measurement of the General Competencies of the Accreditation Council for Graduate Medical Education: A Systematic Review. Academic Medicine, 84(3), 301–309. https://doi.org/10.1097/ACM.0b013e3181971f08 | A Systematic Review | The peer-reviewed literature provides no evidence that current measurement tools can assess the competencies independently of one another. Because further efforts are unlikely to be successful, the authors recommend using the competencies to guide and coordinate specific evaluation efforts, rather than attempting to develop instruments to measure the competencies directly. |
| Patrício MF, Juliao M, Fareleira F, Carniero AV. Is the OSCE a feasible tool to assess competencies in undergraduate medical education? Med Teach. 2013; 35(6):503-514. | Best Evidence Medical Education methodology | The OSCE is a feasible approach to the assessment of clinical competence for use in different cultural and geographical contexts |
| Department of Reproductive Health and Research World Health Organization | Grey Literature | Core competencies in primary care |
| Kylie O'Brien, Peter Hartley, David Dawson, Jason Quick, and Amber Moore. Work readiness in paramedic graduates: what are employers looking for? International Paramedic Practice 2013 3:4, 98-104 | semi-structured, qualitative interviews | This study provides new insight into the expectations and observations of paramedic employers within Australia. The results from this study may inform further educational and professional development opportunities resulting in improvements within the field of paramedicine. |
| Kilner, T. (2004). Desirable attributes of the ambulance technician, paramedic, and clinical supervisor: findings from a Delphi study. Emergency Medicine Journal : EMJ, 21(3), 374–378. https://doi.org/10.1136/emj.2003.008243 | Delphi Study | There was significant agreement among the experts as to the desirable attributes of ambulance staff, many of which do not feature in existing ambulance training curriculums. The findings of this study may therefore be of value in informing future curriculum development and in providing guidance for the selection of staff for each of the occupational groups. |
| O’Brien, K., Moore, A., Hartley, P., & Dawson, D. (2013). Lessons about work readiness from final year paramedic students in an Australian university. Australasian Journal of Paramedicine, 10(4). https://doi.org/10.33151/ajp.10.4.52 | Survey study | Further consideration is needed of how courses will best address the development of clinical and practical skills of students and meet the requirements of ambulance service employers. Collegiate dialogue between employers, educators and students will assist in addressing this. |
| Williams, B., Onsman, A., & Brown, T. (2010). Australian paramedic graduate attributes: a pilot study using exploratory factor analysis. Emergency Medicine Journal : EMJ, 27(10), 794–799. https://doi.org/10.1136/emj.2010.091751 | exploratory factor analysis | It is critical that empirically-based paramedic graduate attributes are developed and agreed upon by both the industry and teaching institutions. Until this occurs, the national standardisation, accreditation and benchmarking of Australian paramedic education programmes will not be possible |
| Włoszczak-Szubzda, A., Jarosz, M. J., & Goniewicz, M. (2013). Professional communication competences of paramedics--practical and educational perspectives. Annals of Agricultural and Environmental Medicine, 20(2), 366–372. | three methods | The results of studies indicate poor efficacy of shaping communication competences of paramedics based on education in the area of general psychology and general interpersonal communication. Communication competences acquired by paramedics during undergraduate education are subject to regression during occupational activity. |
| Royal College of Physicians and Surgeons of Canada | Grey Literature | CanMEDS 2015 Physician Competency Framework |
| Batt, A.M., Hultink, A., Lanos, C., Tierney, B., Grenier, M., Heffern, J. (2021). Advances in Community Paramedicine in Response to COVID-19. Canadian Standards Association, Toronto, ON. | Grey Literature | Advances in Community Paramedicine in Response to COVID-19 |
| Batt, A. M., Williams, B., Brydges, M., Leyenaar, M., & Tavares, W. (2021). New ways of seeing: supplementing existing competency framework development guidelines with systems thinking. Advances in Health Sciences Education : Theory and Practice, 26(4), 1355–1371. https://doi.org/10.1007/s10459-021-10054-x | Conceptual | The ability to represent clinical practice when developing competency frameworks can be improved when features that may be relevant, including their potential interactions, are identifed and understood |
| Choi, B. Y., Blumberg, C., & Williams, K. (2015). Mobile Integrated Health Care and Community Paramedicine: An Emerging Emergency Medical Services Concept. Annals of Emergency Medicine, 67(3), 361–366. https://doi.org/10.1016/j.annemergmed.2015.06.005 | Conceptual | Additional studies are needed to support the clinical and economic benefit of mobile integrated health care and community paramedicine. |
| Dainty, K. N., Seaton, M. B., Drennan, I. R., & Morrison, L. J. (2018). Home Visit‐Based Community Paramedicine and Its Potential Role in Improving Patient‐Centered Primary Care: A Grounded Theory Study and Framework. Health Services Research, 53(5), 3455–3470. https://doi.org/10.1111/1475-6773.12855 | Grounded Theory Study | Home-based community paramedicine programs like EPIC appear to be able to create a patient-centered, safe, responsive therapeutic relationship that is often not possible within the standard primary health care system |
| Eaton, G., Happs, I., & Tanner, R. (2021). Designing and implementing an educational framework for advanced paramedic practitioners rotating into primary care in North Wales. Education for Primary Care, 32(5), 289–295. https://doi.org/10.1080/14739879.2021.1894992 | Semi-structured focus group | Formal research is required to determine any link between provision of education for Advanced Paramedic Practitioners in primary care and patient outcome and safety. |
| Flanagan, B., Warren-James, M., & Young, J. (2023). Evaluation of the CARES Skills Framework as a Peer Support Model in the Paramedic Undergraduate Curriculum: Facilitating Challenging Discussions in a Safe Environment. Prehospital Emergency Care, ahead-of-print(ahead-of-print), 1–7. https://doi.org/10.1080/10903127.2022.2125136 | A modified nominal group technique method was used following a student debriefing session designed to identify problems, generate solutions, and make decisions regarding the efficacy of the CARES skills framework | The normalisation of emotions concerning death and dying was achieved through peer social support. Despite the difficulty in articulating emotions, students expressed that the CARES model provided a secure space for discussing these sensitive topics. During the exercise, students felt acknowledged and connected to their peers, and post-exercise, they reported an improved sense of belonging. |
| Paterson, S. (2023). Preparing pregistration students for lifelong learning and practice. Journal of Paramedic Practice : The Clinical Monthly for Emergency Care Professionals, 15(9), 382–385. https://doi.org/10.12968/jpar.2023.15.9.382 | A short-life working group was formed of members from each university to develop a national practice assessment document (PAD). | Collaborative effort among five Scottish Universities to establish a standardized practice assessment document (PAD) for BSc Paramedic Science programs. |
